# Personalized haemodynamic modelling for non-invasive quantification of arterial stiffness in the human vasculature

**DOI:** 10.1098/rsif.2025.1318

**Published:** 2026-10-14

**Authors:** Xiangdong Zhang, Dan Wu, Fen Miao, Jiajie Zhong, Fan Yu, Xuejiao Zhang, Hao Liu, Ye Li

**Affiliations:** ^1^ Shenzhen Institutes of Advanced Technology Chinese Academy of Sciences, Shenzhen, Guangdong, People’s Republic of China; ^2^ Shenzhen Institutes for Advanced Study UESTC, Shenzhen, Guangdong, People’s Republic of China; ^3^ Futian Center for Chronic Disease Control, Shenzhen, People’s Republic of China; ^4^ Department of Electronics and Mechanical Engineering, Chiba University, Chiba, Japan; ^5^ Shenzhen University of Advanced Technology, Shenzhen, Guangdong, People’s Republic of China

**Keywords:** arterial stiffness, haemodynamics, inverse problem, ultrasound, numerical simulation

## Abstract

Arterial stiffness is a pivotal predictor of cardiovascular events, yet its *in vivo* characterization remains elusive because arterial wall mechanics cannot be directly measured. Conventional inverse approaches require high-resolution imaging to resolve wall motion, restricting their clinical applicability. Here, we present a non-invasive computational framework that infers regional arterial stiffness solely from routinely acquired pressure and blood flow velocity waveforms by leveraging personalized one-dimensional haemodynamic models of the human arterial network. Patient-specific models were constructed for 21 individuals with hypertension using stroke volume, arterial diameters, brachial pressures and carotid flow profiles. Regional stiffness parameters were identified through optimization to reconcile simulated and measured haemodynamics, and validated against brachial-ankle pulse wave velocity. The inferred stiffness demonstrated strong concordance with clinical measurements (*r* = 0.81; standard error = 279 cm s)^–1^. Longitudinal evaluation in 13 patients further revealed therapy-dependent remodelling, with calcium channel blockers selectively reducing pathological stiffening while preserving compliant vessels. Together, these results establish model-based inversion of standard haemodynamic data as a practical, scalable approach for quantifying arterial stiffness, enabling individualized cardiovascular risk assessment and treatment monitoring.

## 1. Introduction

Arterial stiffness critically influences cardiovascular function and is a key predictor of cardiovascular events. Numerous clinical studies have demonstrated a correlation between increased arterial stiffness and various cardiovascular conditions, including hypertension [1–3], heart diseases [4,5], stroke [6] and diabetes [7]. Increased central or aortic arterial stiffness leads to elevated systolic blood pressure (SBP) and pulse pressure (PP, systolic minus diastolic pressures) owing to decreased compliance with pulsatile blood flow [2]. Elevated SBP and PP are harmful to vital organs and can result in heart failure [4], coronary artery diseases [5] and kidney failure [8]. In addition, high PP is a recognized risk factor for stroke [6]. Additionally, regional arterial stiffness, such as in the carotid and femoral arteries, correlates with cardiovascular diseases [9,10]. Therefore, the quantitative assessment of arterial stiffness holds significant clinical value.

Increased arterial stiffness will lead to increased pulse wave velocity (PWV). Measurement of PWV is the most commonly used method for assessing arterial stiffness quantitatively. In clinical applications, PWV is typically measured non-invasively by probing two superficial arterial sites and is calculated by dividing the arterial path length by the pulse wave transit time (PTT) obtained from sensors. By placing pressure sensors on the common carotid artery and the femoral artery, the carotid-femoral PWV (cf-PWV) can be calculated, which is commonly used to estimate aortic PWV [11]. However, because these sensors require skilled operators to locate the arteries, more automated methods have been developed, such as brachial-ankle PWV (ba-PWV) [12] and finger-toe PWV [13], which place fixed sensors on more peripheral sites. While these indices are easier to measure, they are less strongly correlated with aortic stiffness owing to the involvement of more peripheral arteries. Moreover, these indices do not directly quantify ascending aortic stiffness.

In recent decades, magnetic resonance imaging (MRI)-based techniques have emerged as methods for direct measurement of PWV in the aorta [14,15]. However, the accuracy of these measurements is limited by the low frame rate of current MRI machines, and the associated costs are high. In addition to direct PWV measurement, indirect methods based on machine learning techniques are also being explored [16,17]. However, the accuracy of these data-driven methods still requires improvement, particularly for patient-specific measurements [18].

Measurement of local arterial stiffness has also been widely studied, as it may enable a more precise evaluation of local vascular function. Several ultrasound-based methods have been developed in recent decades. The pulse wave imaging method directly tracks wave propagation along arterial walls using ultrasound images with ultra-high frame rates [19,20]. The ultrasound elastography method estimates the shear modulus of arterial walls by analysing the perturbed shear waves in tissues [21,22]. A common limitation of these methods is also a general limitation of ultrasound measurements: it is difficult to acquire high-quality signals from deep arteries. As a result, most studies have been performed on carotid arteries. Magnetic resonance elastography [23,24] can overcome some of these limitations.

Owing to the challenges in directly measuring the elastic properties of arterial walls, recent studies have combined medical images with computational mechanics models to identify arterial stiffness inversely. Farzaneh *et al.* [25,26] estimated local stiffness around an ascending thoracic aortic aneurysm using solid mechanics models. By combining local strains measured from computed tomography (CT) images with local equilibrium equations of an elastic elliptical tube, they were able to map local arterial stiffness around the aneurysm. A strong correlation was found between the stiffness of the aneurysm and the rupture risk criterion in their study. Mousavi *et al.* [27] developed a multi-layer model of arterial walls, incorporating elastin, collagen fibres and smooth muscle cells, to estimate the stiffness of different wall components from CT-measured strains. Their results indicated that the loss of elastin may predict the morphological progression of an ascending thoracic aortic aneurysm. Narayanan *et al.* [28] reconstructed a heterogeneous, hyperelastic arterial wall model from intravascular optical coherence tomography (OCT) image series, discretizing the model into unstructured three-dimensional finite element meshes. Using a multi-step genetic optimization algorithm, they tuned the elastic parameters to minimize errors between simulated and OCT-measured arterial wall displacements, and their results demonstrated good reproducibility.

Inverse methods based on solid mechanics models rely on accurate measurement of arterial wall displacements, which typically require high spatial resolution, making it challenging to measure smaller vessels non-invasively. Fluid-structure interaction models offer an alternative approach, allowing blood flow velocities to be used for estimating arterial stiffness inversely. The reduced-order one-dimensional haemodynamic model for arteries [29–32] is a widely studied and effective approach for simulating blood velocity waveforms, blood pressure waveforms, average wall displacements and PWV [33]. McGarry *et al.* [34] estimated arterial stiffness from ultrasound-measured wall displacements and flow velocities based on inverse analysis of a one-dimensional haemodynamic model. This method was validated using single silicone tubes with known distributions of wall stiffness. However, it still primarily relies on accurate measurements of arterial wall displacements, and it does not account for the effects of complex wave propagation and reflections in artery networks on velocity waveforms.

The shape of blood flow waveforms is influenced by the arterial stiffness of both the local vessel and the entire arterial network. Different-shaped waveforms can be simulated precisely using personalized one-dimensional haemodynamic models of the human cardiovascular system, as demonstrated in our previous study [35].

In this study, we propose an inverse method to infer arterial stiffness from measured velocity waveforms and blood pressures based on personalized one-dimensional haemodynamic modelling of the human cardiovascular system. The inferred stiffness is validated by comparing it to measured ba-PWV. Additionally, we apply the proposed method to track variations in arterial stiffness in hypertensive patients and investigate the effects of different antihypertensive agents. The proposed method is entirely non-invasive, low-cost and does not require resolving arterial wall motion. It only requires a standard medical ultrasound system and a blood pressure monitor.

## 2. Material and methods

### 2.1. A one-dimensional haemodynamic model

The personalized one-dimensional haemodynamic modelling method in this study was developed based on our previously validated work [35], in which the dynamics of major arteries in the human cardiovascular system are modelled as one-dimensional incompressible viscous flow coupled with linear elastic arterial walls. The governing equations of a single artery segment are given in [36–38]:

(2.1a)∂U∂t+∂∂z(U22+Pρ)+8πνUA=0,(2.1b)∂A∂t+∂∂z(UA)=0(2.1c)and P−P0=4Eh3r0(AA0−1),

where the three variables are the average velocity U, pressure P and the cross-sectional area A. In the linear elastic equation (2.1*c*), the reference pressure $P_{0}$ corresponds to the pressure when the area is $A_{0}$ (with radius $r_{0}$). The parameter ρ represents the constant blood density, with $\rho =1.06\;{\text{g cm}}^{-3}$ used in this study. The friction parameter 8πν is based on the assumption that the blood velocity profile is parabolic [39], where ν is the blood viscosity and $\nu =4.43\;{\text{s}}^{-1}{\text{cm}}^{2}$. The parameter E denotes the elastic modulus of the arterial wall, and h is the wall thickness. Because h is difficult to measure accurately, we use the product of E and h as a single parameter, and we use the term ‘arterial stiffness’ to refer to Eh in this study.

To simulate the human cardiovascular system, we use a one-dimensional artery network that includes the major human arteries, as shown in figure 1a. The volumetric flow rates (Q=UA) are conserved at the bifurcations in the artery network, and continuous blood pressure is assumed. At the inlet of the network (ascending aorta), the prescribed volumetric flow rate $Q_{\text{inlet}}(t)$ constructed using periodic piecewise cosine functions is applied as follows:

(2.2) $$\begin{array}{rl} & Q_{\text{inlet}}(t)=\left\{ \begin{array}{l} Q_{\text{max}}(1-cos(\pi t/t_{1}))/2,\;t\in [0,t_{1}),\\ Q_{\text{max}}\text{cos}(\pi (t-t_{1})/(2t_{2}-2t_{1})),\;t\in [t_{1},t_{2}),\\ -\chi Q_{\text{max}}\text{sin}(\pi (t-t_{2})/(2\chi t_{2}-2\chi t_{1})),\;t\in [t_{2},t_{3}),\\ -\chi Q_{\text{max}}(1+\text{cos}(\pi (t-t_{3})/(t_{4}-t_{3})))/2,\;t\in [t_{3},t_{4}),\\ 0,\;t\in [t_{4},T]. \end{array}\right. \end{array}$$

Here, $Q_{\text{max}}$ is the maximum flow rate, χ is the backflow ratio where $-\chi Q_{\text{max}}$ is the maximum backflow rate and $t_{3}-t_{2}=\chi (t_{2}-t_{1})$. The cardiac cycle period is denoted by T. The integral of the positive flow rates over one period equals the stroke volume (SV):

(2.3) $$\text{SV}=\frac{t_{1}}{2}Q_{\text{max}}+\frac{2(t_{2}-t_{1})}{\pi }Q_{\text{max}}.$$

**Figure 1.**
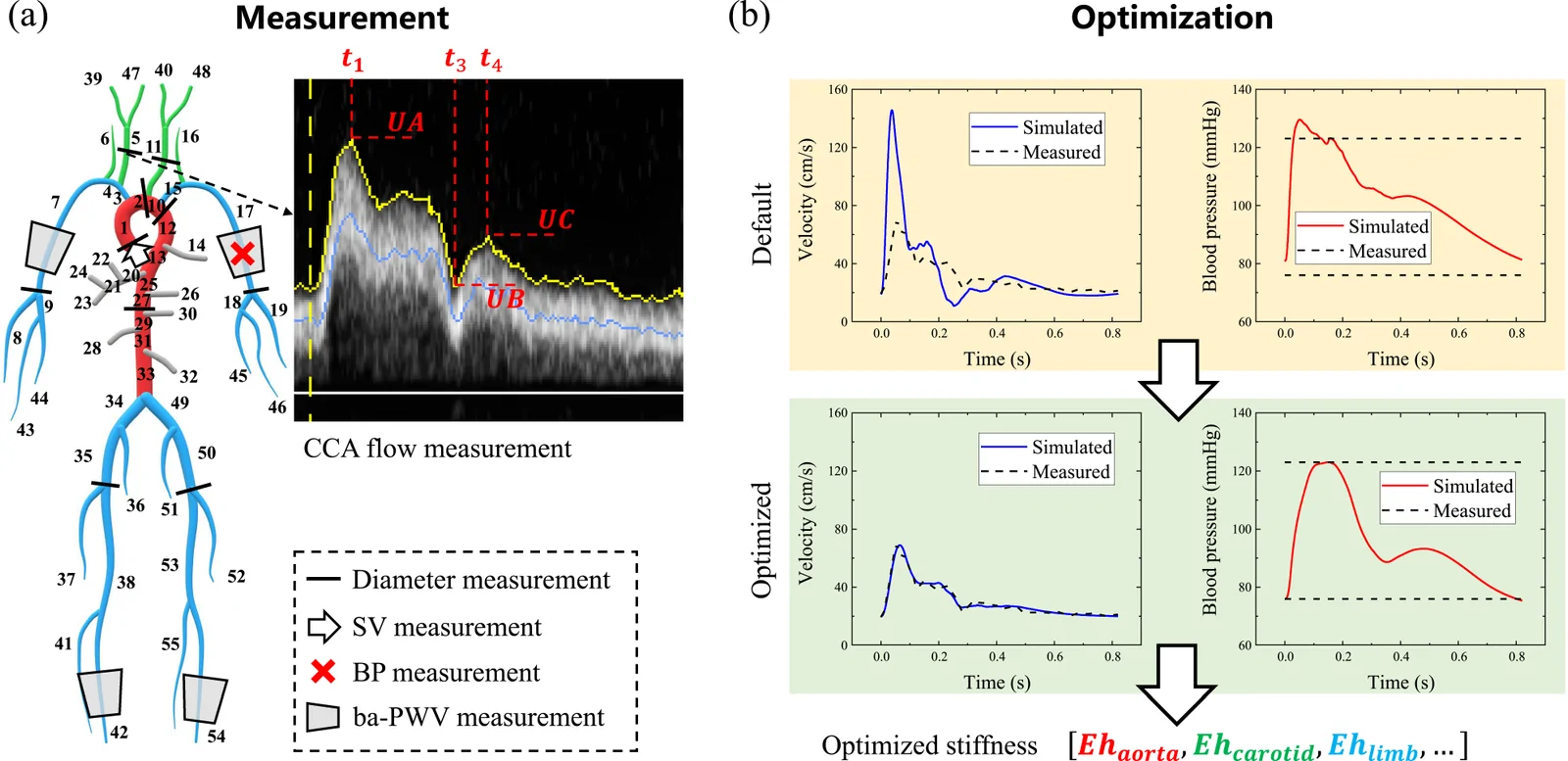
Personalized one-dimensional haemodynamic modelling of the human arterial system: (a) the arterial tree with partitioned clusters in different colours and measurements of different data; (b) the concept of the optimization algorithm to get personalized arterial stiffness in different arterial clusters.

The zero-dimensional RCR Windkessel models [40] are coupled with the one-dimensional models at the network outlets, with zero distal pressures. The one-dimensional models are discretized using the finite difference method, and the governing equations are solved using the MacCormack scheme. An efficient coupling algorithm based on the ghost point method [35] is implemented to couple the one-dimensional models with the inlet boundary conditions and the outlet zero-dimensional models. Further details of the numerical schemes can be found in [35].

### 2.2. Personalized modelling of the human arterial system

To determine the parameters of the one-dimensional model for different individuals, such as the reference radius and arterial stiffness (Eh) at various locations, we developed a personalized modelling method based on ultrasound and blood pressure measurements, as illustrated in figure 1, where each artery cluster is colour-coded in figure 1a. The diameters of several key arterial locations are measured using ultrasound, and the diameters of all artery segments within the same cluster are scaled according to the ratios between the measured diameters and the initial diameters; for example, the left lower limb segments in the limb cluster (blue) are scaled using the distal diameter of the left external iliac artery. The initial parameters of the artery tree model are listed in appendix A, based on the literature data from [37,41]. Data from [31] for the lower limb segments are used to replace the overestimated distal diameters in [37,41]. We measured diameters of the aorta (red) at the ends of four segments, as shown in figure 1a. For the segment ends between these measurement locations, linear interpolation was used to estimate the diameters based on the distances from the nearest measured locations. For segments 29, 31 and 33, their diameters are uniformly scaled using the ratio between the measured and initial distal diameters of segment 27. For the small side branches on the aorta, their initial diameters and stiffness are kept unchanged for each patient. The lengths of all segments are uniformly scaled based on the same data source, using the ratio of the subject’s height to 175 cm.

Although the tapering diameter data from [31] are used for the lower limbs, the initial stiffness should also be calibrated to reproduce reasonable ba-PWV. According to a report, the average PWV of femoral arteries is about 10.58 m s^–1^ [42], but the simulated PWV of femoral arteries with default parameters is about 7.38 m s^–1^, which would lead to systematic underestimation of ba-PWV. Therefore, we calibrated (doubled) the default $E_{0}$ of the lower limb segments based on the Moens–Korteweg equation [43] ${\text{PWV}}^{2}=Eh/(2r_{0}\rho )$:

(2.4) $$E=\frac{{\text{PWV}}^{2}}{{\text{PWV}}_{0}^{2}}E_{0}\approx 2E_{0},$$

and the calibrated E is listed in parentheses for each lower limb segment in Appendix A.

SV and blood flow waveforms in the right common carotid artery (CCA) are also recorded using ultrasound. Additionally, the inlet parameters $t_{1}$, $t_{3}$ and $t_{4}$ are measured from the CCA waveform, and the backflow ratio χ is estimated by χ=(UC−UB)/UA, where UA denotes the peak systolic velocity, UB denotes the notch velocity and UC is the peak velocity after $t_{3}$, as illustrated in the graph. Systolic blood pressure (SBP) and diastolic blood pressure (DBP) are measured at the left brachial artery.

Following these measurements, the arterial stiffness (Eh) for different arterial regions, as well as the outlet resistance (R) and compliance (C), remain unknown. Therefore, we inversely estimated these parameters using the measured flow waveforms and blood pressures, as shown in figure 1b, based on the Levenberg–Marquardt (LM) optimization algorithm. The objective function for the optimization was defined as follows:

(2.5a)Fm=m.s.e.+α(SBPs−SBPm)2+α(DBPs−DBPm)2+β(UAs−UAm)2+β(UBs−UBm)2(2.5b)and m.s.e.=1n∑i=1n[UCCAm(i)−UCCAs(i)]2,

where m.s.e. is the mean squared error between the simulated CCA flow waveforms (cm s^–1^) and the measured waveform, UCCA denotes the velocity of the CCA flow and n denotes the number of sample points on the measured flow waveforms. The subscript s denotes simulated results and m denotes measured results. The simulated SBP and DBP (mm Hg) are set as the maximum and the minimum of the simulated pressure waves on 2/3 of the length of the left brachial artery from the proximal end, as shown in figure 1. The parameters α and β are weights of the errors. In this study, α=β=1.0 is used because the four squared errors are in the same magnitude, and all errors get well-converged results with them. Then, the LM optimization algorithm is constructed as follows:

(2.6a)[JkTJk+λdiag(JkTJk)]ΔSk=JkTΔYk(2.6b)and Sk+1=Sk+ΔSk.

Here, k denotes the iteration step and the iteration is stopped if

(2.7) $$\|\Delta S_{k}\|<\gamma ,$$

where γ=0.05 in this study and ‖⋅‖ is the $l^{2}$ norm of a vector. The components of vector $\Delta Y=(\Delta y_{1},\Delta y_{2},...\Delta y_{5n}{)}^{T}$ in equation (2.6) are defined as:

(2.8) $$\begin{array}{rl} & \Delta y_{i}=\left\{ \begin{array}{l} UCCA_{m}(i)-UCCA_{s}(i),\;1\leq i\leq n,\\ {\text{SBP}}_{m}-{\text{SBP}}_{s},\;n+1\leq i\leq 2n,\\ {\text{DBP}}_{m}-{\text{DBP}}_{s},\;2n+1\leq i\leq 3n,\\ UA_{m}-UA_{s},\;3n+1\leq i\leq 4n,\\ UB_{m}-UB_{s},\;4n+1\leq i\leq 5n. \end{array}\right. \end{array}$$

Then, it is easy to get:

(2.9) $$F_{m}=\frac{\|\Delta Y{\|}^{2}}{n}.$$

Therefore, the proposed algorithm aims to minimize the function $F_{m}$. In equation (2.6), ΔS denotes the change of the parameter set $S=(s_{1},\ldots s_{p})$ in each iteration, where p=7 and the set includes:

(2.10) $$S={\left(C_{Eh-\text{aorta}},C_{Eh-\text{carotid}},C_{Eh-\text{limb}},C_{R-\text{carotid}},C_{C-\text{carotid}},C_{R},C_{C}\right)}^{T}.$$

The parameter set includes seven scaling factors (the ratio of the subject-specific magnitude to the baseline magnitude) from the initial data in table 3, where the proximal resistance $R_{p}$, distal resistance $R_{d}$ and compliance C for each outlet are also listed in the table. The scaling factor $C_{Eh-\text{aorta}}$ is the factor of the aorta cluster’s (red in figure 1a) stiffness Eh, $C_{Eh-\text{carotid}}$ is for the carotid cluster (green), $C_{Eh-\text{limb}}$ is for the limb cluster (blue), $C_{R-\text{carotid}}$ is the factor of the carotid cluster’s distal resistance $R_{d}$, $C_{C-\text{carotid}}$ is the factor of the carotid cluster’s compliance, $C_{R}$ is the uniform resistance coefficient for $R_{d}$ except the carotid cluster and $C_{C}$ is the uniform peripheral compliance coefficient (except carotid cluster). The proximal resistance $R_{p}$ for each one-dimensional outlet is recalculated in each simulation using the outlet properties after scaling: $R_{p}=\rho \cdot \text{PWV}/A$ [44]. To get unique optimized solutions, the parameters should have different effects on CCA waveforms and blood pressures. Analysis in the appendix has revealed that these parameters are independent to each other, so the risk of multiple local solutions can be reduced.

**Table 1.** Therapy plans.

number of data sets k	number of patients	ARB/ACEI	CCB	CCB+ARB/ACEI
1	8	4	3	1
2	8	2	4	2
3	5	0	3	2

**Table 2.** Optimized parameters.

	$C_{Eh-\text{aorta}}$	$C_{Eh-\text{carotid}}$	$C_{Eh-\text{limb}}$	$C_{R-\text{carotid}}$	$C_{C-\text{carotid}}$	$C_{R}$	$C_{C}$
max	9.08	9.43	2.37	1.41	5.71	4.20	4.97
min	0.75	1.60	0.39	0.27	0.38	1.18	0.21
average	2.99	3.35	0.95	0.85	1.19	2.45	1.65

**Table 3.** Initial parameters of the artery tree before scaling.

								$R_{p}$	$R_{d}$	C
ID	L (cm)	$r_{p}$ (cm)	$r_{d}$ (cm)	E (MPa)	h (cm)	link1	link2	$\;(\text{mm Hg}{\text{s ml}}^{-1})$	$\;(\text{mm Hg}{\text{s ml}}^{-1})$	$\;({\text{ml mm Hg}}^{-1})$
1	6	1.525	1.42	0.4	0.1	2	3	0	0	0
2	3	1.42	1.342	0.4	0.1	10	11	0	0	0
3	3.5	0.65	0.62	0.4	0.08	4	5	0	0	0
4	3.5	0.425	0.407	0.4	0.067	6	7	0	0	0
5	17.7	0.4	0.37	0.4	0.063	39	47	0	0	0
6	13.5	0.2	0.2	0.8	0.045	0	0	6.1	27.87	0.0126
7	39.8	0.407	0.23	0.4	0.067	8	9	0	0	0
8	22	0.175	0.14	0.8	0.043	0	0	14.21	18.34	0.0143
9	6.7	0.215	0.215	0.8	0.046	43	44	0	0	0
10	4	1.342	1.246	0.4	0.1	12	15	0	0	0
11	20.8	0.4	0.37	0.4	0.063	40	48	0	0	0
12	5.5	1.246	1.124	0.4	0.1	13	14	0	0	0
13	10.5	1.124	0.924	0.4	0.1	20	25	0	0	0
14	7.3	0.3	0.3	0.4	0.049	0	0	2	6.04	0.0542
15	3.5	0.425	0.407	0.4	0.066	16	17	0	0	0
16	13.5	0.2	0.2	0.8	0.045	0	0	6.1	27.87	0.0126
17	39.8	0.407	0.23	0.4	0.067	18	19	0	0	0
18	6.7	0.215	0.215	0.8	0.046	45	46	0	0	0
19	22	0.175	0.14	0.8	0.043	0	0	14.21	18.34	0.0143
20	2	0.35	0.3	0.4	0.064	21	22	0	0	0
21	2	0.3	0.25	0.4	0.064	23	24	0	0	0
22	6.5	0.275	0.25	0.4	0.049	0	0	2.8	17.48	0.0208
23	5.8	0.175	0.15	0.4	0.054	0	0	8.59	22.97	0.0139
24	5.5	0.2	0.2	0.4	0.045	0	0	4.05	9.59	0.0325
25	5.3	0.924	0.924	0.4	0.1	26	27	0	0	0
26	5	0.4	0.35	0.4	0.069	0	0	1.2	4.15	0.081
27	1.5	0.838	0.814	0.4	0.1	28	29	0	0	0
28	3	0.275	0.275	0.4	0.053	0	0	2.02	4.64	0.0667
29	1.5	0.814	0.792	0.4	0.1	30	31	0	0	0
30	3	0.275	0.275	0.4	0.053	0	0	2.02	4.64	0.0667
31	6	0.792	0.627	0.4	0.1	32	33	0	0	0
32	3.8	0.2	0.175	0.4	0.043	0	0	5.37	33.1	0.011
33	6	0.627	0.55	0.4	0.1	34	49	0	0	0
34	5.8	0.6	0.6	0.4(0.8)	0.06	35	36	0	0	0
35	14.5	0.56	0.5	0.8(1.6)	0.053	37	38	0	0	0
36	4.5	0.35	0.3	1.6(3.2)	0.04	0	0	6.4	25.28	0.0136
37	11.3	0.34	0.32	0.8(1.6)	0.047	0	0	4.99	14.4	0.0226
38	44.3	0.41	0.33	0.8(1.6)	0.05	41	42	0	0	0
39	17.7	0.2	0.2	0.8	0.042	0	0	5.23	23.53	0.0148
40	17.6	0.3	0.275	0.8	0.045	0	0	2.53	25.42	0.0148
41	34.4	0.16	0.1	1.6(3.2)	0.045	0	0	9.9	32.78	0.0102
42	32.2	0.23	0.1	1.6(3.2)	0.039	0	0	3.96	15.11	0.0226
43	7	0.1	0.1	1.6	0.028	0	0	39.43	424.01	0.0009
44	17	0.203	0.18	0.8	0.046	0	0	10.12	21.2	0.0143
45	17	0.203	0.18	0.8	0.046	0	0	10.12	21.2	0.0143
46	7	0.1	0.1	1.6	0.028	0	0	39.43	424.01	0.0009
47	17.6	0.3	0.275	0.8	0.045	0	0	2.53	25.42	0.0148
48	17.7	0.2	0.2	0.8	0.042	0	0	5.23	23.53	0.0148
49	5.8	0.6	0.6	0.4(0.8)	0.06	50	51	0	0	0
50	14.5	0.56	0.5	0.8(1.6)	0.053	52	53	0	0	0
51	4.5	0.35	0.3	1.6(3.2)	0.04	0	0	6.4	25.28	0.0136
52	11.3	0.34	0.32	0.8(1.6)	0.047	0	0	4.99	14.4	0.0226
53	44.3	0.41	0.33	0.8(1.6)	0.05	54	55	0	0	0
54	32.2	0.16	0.1	1.6(3.2)	0.045	0	0	9.9	32.78	0.0102
55	34.4	0.23	0.1	1.6(3.2)	0.039	0	0	3.96	15.11	0.0226

The Jacobian matrix J in equation (2.6) is a 5n×p matrix, and it is calculated numerically using the finite difference method:

(2.11) $$\begin{array}{rl} & J_{ij}=\left\{ \begin{array}{l} (UCCA_{s^{*}}(i)-UCCA_{s}(i))/(0.1s_{j}),\;1\leq i\leq n,\\ ({\text{SBP}}_{s^{*}}-{\text{SBP}}_{s})/(0.1s_{j}),\;n+1\leq i\leq 2n,\\ ({\text{DBP}}_{s^{*}}-{\text{DBP}}_{s})/(0.1s_{j}),\;2n+1\leq i\leq 3n,\\ (UA_{s^{*}}-UA_{s})/(0.1s_{j}),\;3n+1\leq i\leq 4n,\\ (UB_{s^{*}}-UB_{s})/(0.1s_{j}),\;4n+1\leq i\leq 5n, \end{array}\right. \end{array}$$

where the subscript $s^{*}$ denotes simulated results with the parameter set $S^{*}=(s_{1},\ldots 1.1s_{j},\ldots s_{p}{)}^{T}$ in which the j-th parameter is perturbed to 110% of the original value. The damping coefficient λ equals $10^{-3}$ by default, which balances numerical stability and computing speed well for most cases. A smaller λ will lead to oscillations of the solutions, and a large λ makes the update similar to a slow gradient-descent method. For the unstable cases (about 30%), λ=0.1 is used to enhance the numerical stability.

### 2.3. Simulations of pulse wave transit times and pulse wave velocities

PTTs and PWVs can also be calculated from the simulated results. The PTT between two arterial locations can be calculated as the time difference between the two end-diastolic times on simulated pressure waveforms, as shown in figure 2. In this study, we consider two types of PWVs: the ba-PWV and the cf-PWV. The ba-PWV is calculated using the formula [45]:

(2.12) $$\text{ba-PWV}=\frac{5.934\times \text{height(cm)}+144.01}{\text{ba-PTT(ms)}},$$

which is the same as most ba-PWV measurement devices’ formulas. The calculation of ba-PTT is shown in figure 2a.

**Figure 2.**
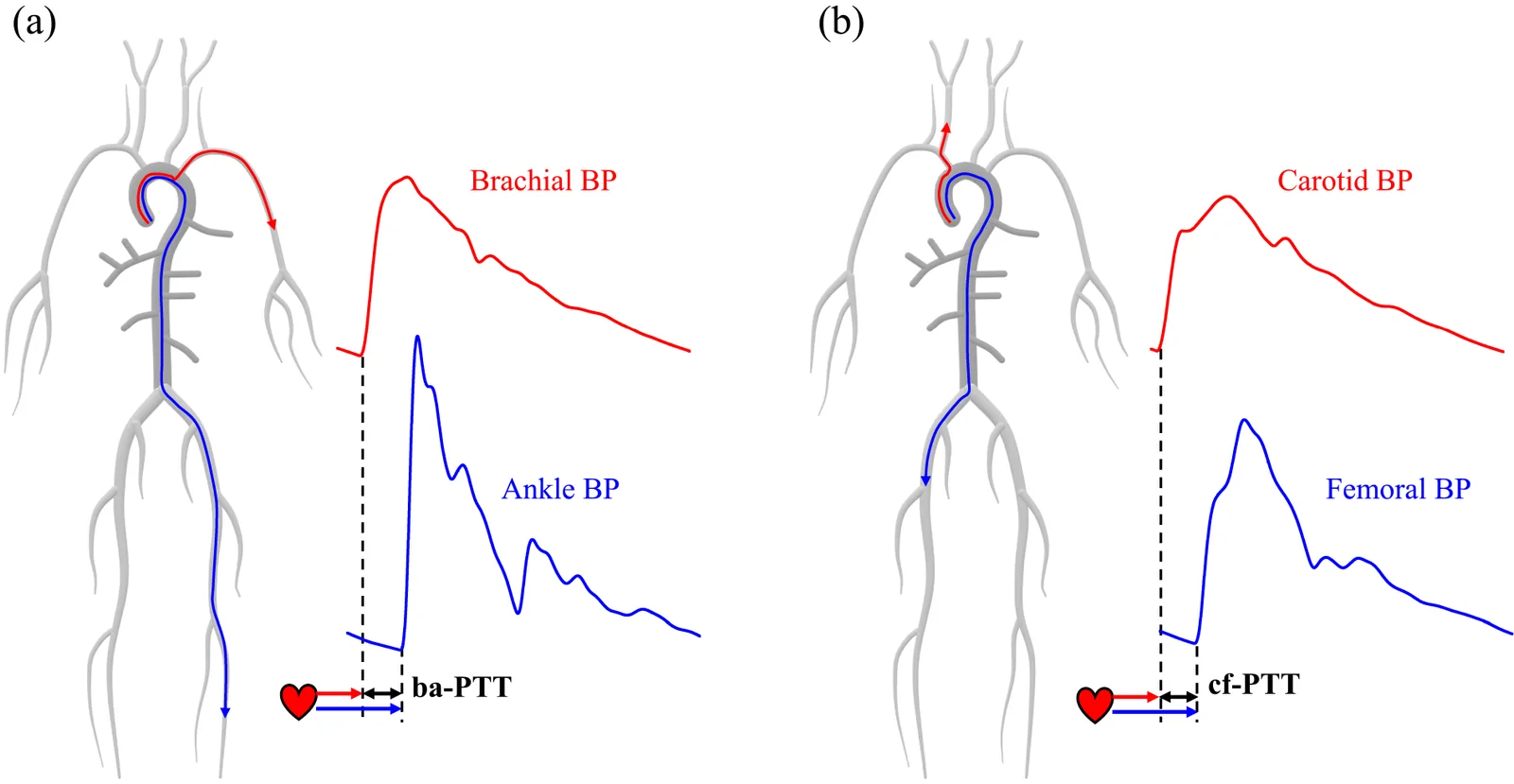
Simulations of (a) ba-PTT and (b) cf-PTT.

The cf-PWV is calculated by:

(2.13) $$\text{cf-PWV}=\frac{L_{hf}-L_{hc}}{\text{cf-PTT}},$$

where $L_{hf}$ is the arterial path length from the heart to the femoral artery, $L_{hc}$ is the path length from the heart to the common carotid artery, and the cf-PTT calculation is illustrated in figure 2b. In practice, the two lengths are usually estimated from ruler measurements on the body surface with a correction factor of 0.8 [46].

### 2.4. Physiological data of patients with hypertension

In this study, we developed personalized one-dimensional models for 21 patients with hypertension based on totally non-invasive measurements. The physiology data were collected in the Xinhua Community Health Center in Shenzhen, People’s Republic of China. We recruited volunteers from patients who visited the health centre for antihypertensive treatment. The primary inclusion criterion was that the patient was about to begin or change antihypertensive medications, as we aimed to track significant changes in haemodynamic parameters within the same subject. This study is observational, and no interventions were made to the therapy plans. Patients who were pregnant, had diabetes, cancer or mental disorders were excluded. Prior to the measurements, we recorded the patients’ height and therapy plans. In this study, we analyse the effects of three major types of antihypertensive agents, either individually or in combination: calcium channel blockers (CCBs), angiotensin- converting enzyme inhibitors (ACEIs) and angiotensin receptor blockers (ARBs). CCBs reduce calcium influx into muscle cells, thereby decreasing muscle contraction [43], which may affect the stiffness of large vessels. ACEIs and ARBs can reduce peripheral vascular resistance [47]. Patients using other types of antihypertensive agents were excluded. To monitor changes in model parameters after medication use, we encouraged patients to return to the centre twice every two weeks to measure their physiological data. By the end of the study, five patients returned twice, eight returned once and eight never returned, as shown in table 1. The therapy plans are also listed in the table.

Ultrasound measurements were performed using the Resona 7 Diagnostic Ultrasound System (Mindray Medical International Limited, People’s Republic of China) by an experienced physician. Diameters were measured in B-mode, stroke volumes in M-mode and flow waveforms in Doppler mode. The diameters were measured only once, as they remain relatively constant over a short period [48].

To validate the personalized haemodynamic models, we also measured the ba-PWV of each patient using the Omron BP-203RPEIII (Omron Corporation, Japan) at each visit. The device measures ba-PTT and calculates ba-PWV automatically using equation (2.12), based on the pressure waveforms recorded from four pressure cuffs. The left brachial blood pressure is recorded simultaneously from one of the cuffs. Since the pressurized cuffs may temporarily alter the haemodynamic state, ba-PWV measurements were performed after the ultrasound measurements to avoid an additional recovery period. To maintain haemodynamic equilibrium, the subjects remained lying on the same bed throughout the procedure. It took approximately 30 minutes to finish all the measurements for each patient during the initial visit and 15 minutes during follow-up measurements.

## 3. Results

### 3.1. Convergence of the optimization algorithm

The proposed optimization algorithm yields converged model parameters, CCA flow waveforms, blood pressures and PTTs for each patient. Figure 3 shows the simulated results for a patient during the optimization procedure. The figure shows close agreement between the optimized and measured flow waveforms. Additionally, the blood pressure and PTT values converge within several iterations. For all simulation cases, the average number of iterations is 10 and the average computing time is 592 s using a single thread (Intel(R) Xeon(R) Silver 4114 CPU @ 2.20GHz). The average m.s.e. of the CCA waveforms across all personalized models is 4.09 ${\text{cm}}^{2}{\text{s}}^{\text{--}2}$, and the blood pressure errors remain below 1.0 mm Hg for all models. Figure 4 presents typical CCA flow waveforms for different patient types, with the well-fitted waveforms illustrating the adaptability of the one-dimensional haemodynamic model. Table 2 shows summary statistics of the optimized parameters. As shown in the table, the average aortic stiffness and carotid stiffness are higher than the reference value, while the limb stiffness is close to the reference value. These results are consistent with the ageing pattern of human arteries [49].

**Figure 3.**
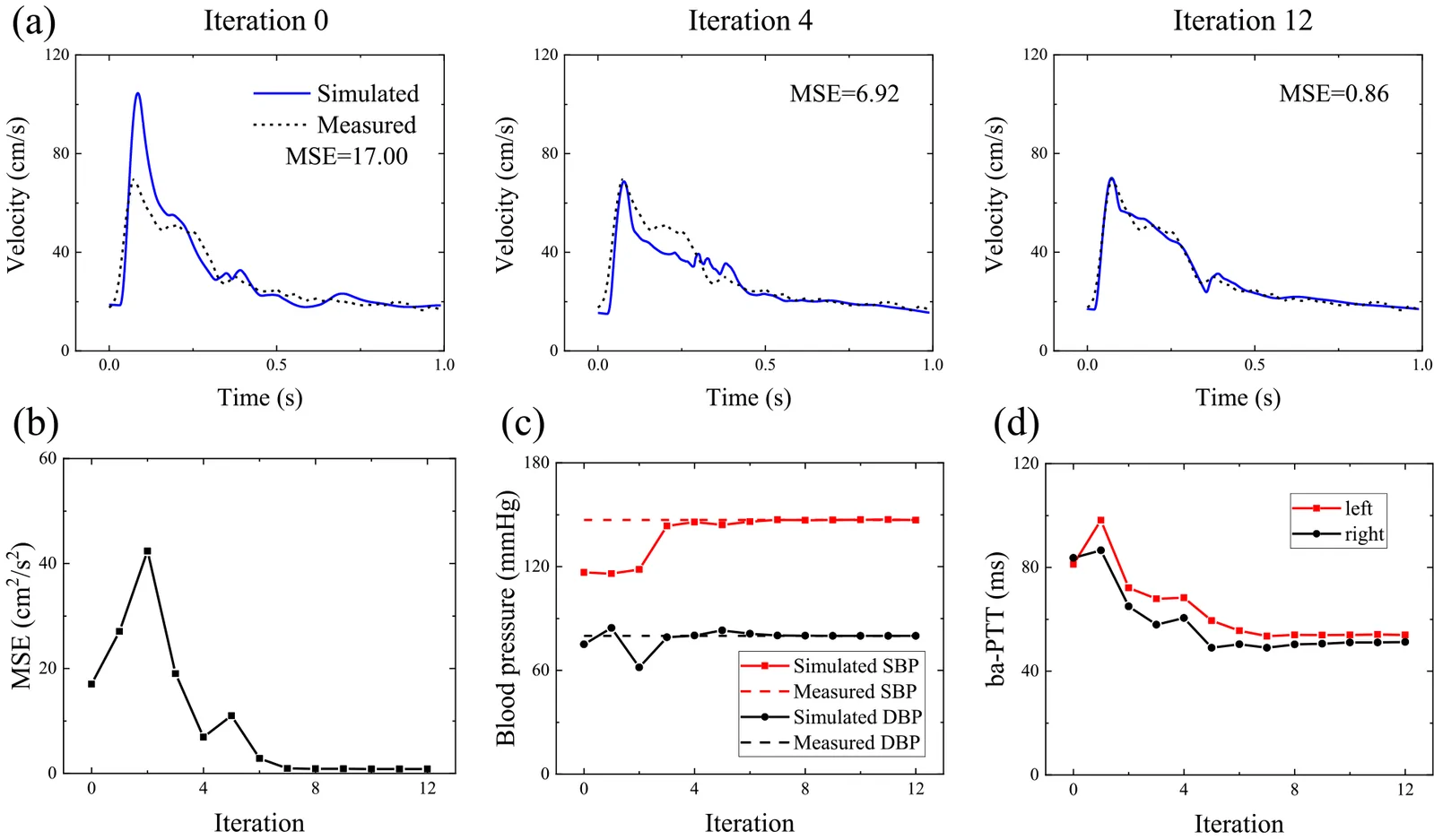
Convergence of the simulated results: (a) calibrated CCA flow velocity waveforms, (b) m.s.e. of the simulated CCA flow waveforms, (c) simulated SBP and DBP, and (d) simulated left and right ba-PTTs.

**Figure 4.**
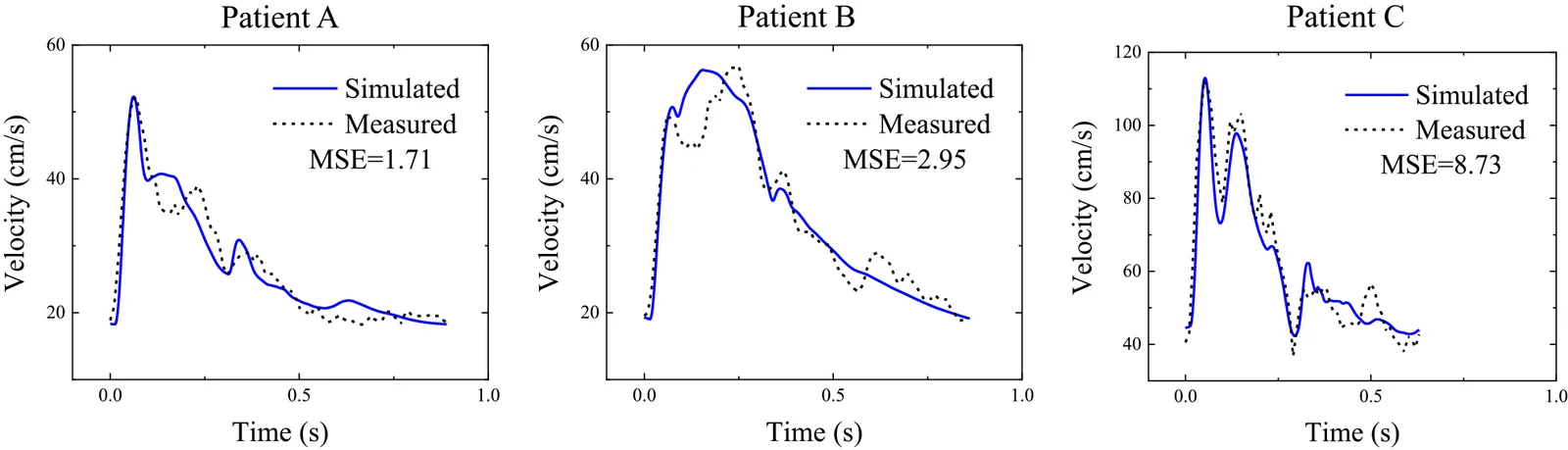
Typical calibrated CCA flow waveforms from three patients.

### 3.2. Quantification of arterial stiffness

The predicted average ba-PTTs and ba-PWVs from the personalized models are compared with the measured average ba-PTTs and ba-PWVs in figure 5. As shown in the figure, the correlation coefficient is 0.84 for the ba-PTTs and 0.81 for the ba-PWVs. The standard error is 9.2 ms for ba-PTTs and 279 cm s^–1^ for ba-PWVs. Systematic errors may originate from the uniform scaling of arterial lengths and the fixed cuff location (2/3 from the proximal end) of the brachial/ankle cuff, potentially shifting the regression line from the line of identity (y=x). Besides, measurement errors may also deviate the predictions.

**Figure 5.**
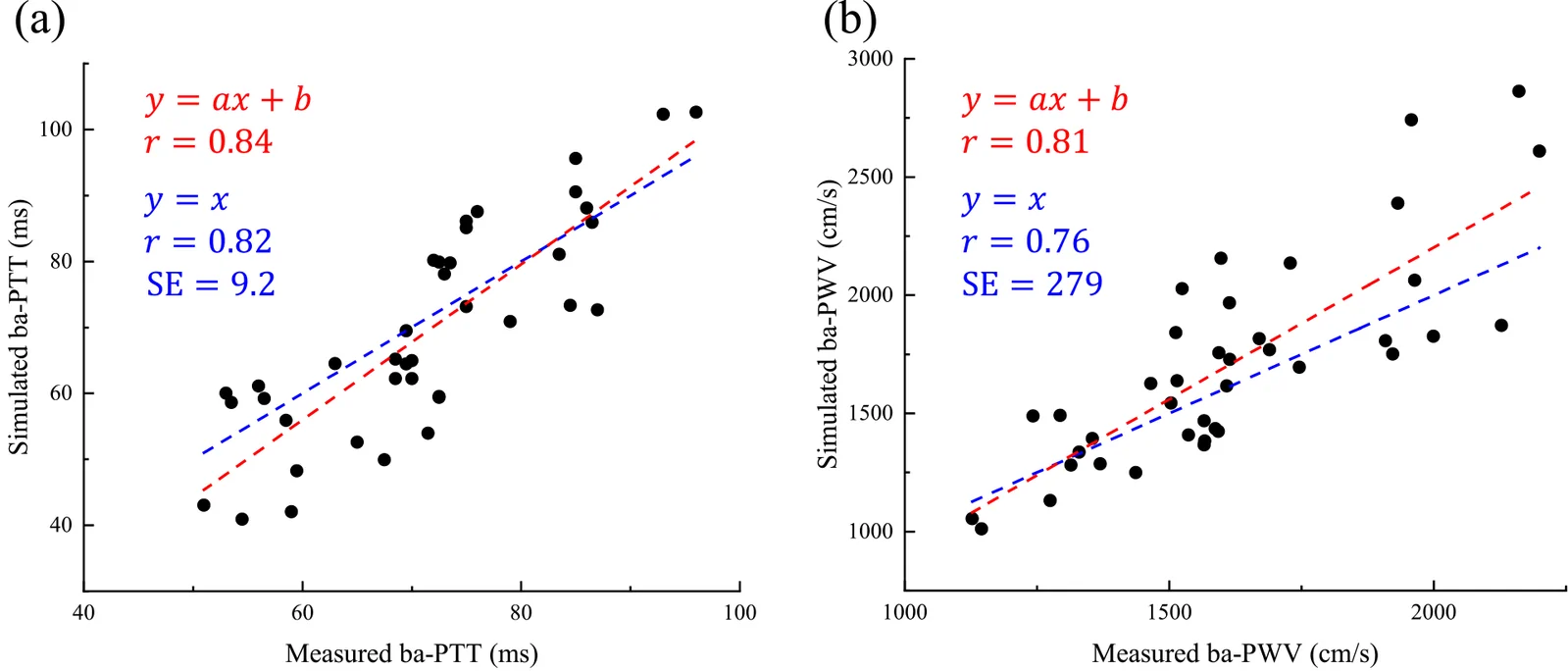
Comparisons between (a) simulated ba-PTT and measured ba-PTT, and (b) simulated ba-PWV and measured ba-PWV.

The quantitative evaluation of aortic stiffness holds significant clinical value. Therefore, we compared the optimized aortic stiffness coefficient $C_{Eh-\text{aorta}}$ with different types of PWVs, as shown in figure 6. According to the MK equation, arterial stiffness (Eh) is proportional to the square of the local PWV. Thus, a parabolic fit was performed between $C_{Eh-\text{aorta}}$ and PWVs. The measured ba-PWVs are compared with $C_{Eh-\text{aorta}}$ in figure 6a, yielding a correlation coefficient of 0.78. Some data points with large deviations are expected as ba-PWV is sensitive to the stiffness of both the aorta and the limb arteries, while $C_{Eh-\text{aorta}}$ represents only the aortic stiffness. By contrast to ba-PWV, cf-PWV primarily reflects aortic stiffness. Therefore, a significant correlation (*r* = 0.94) is observed between the simulated cf-PWV and $C_{Eh-\text{aorta}}$.

**Figure 6.**
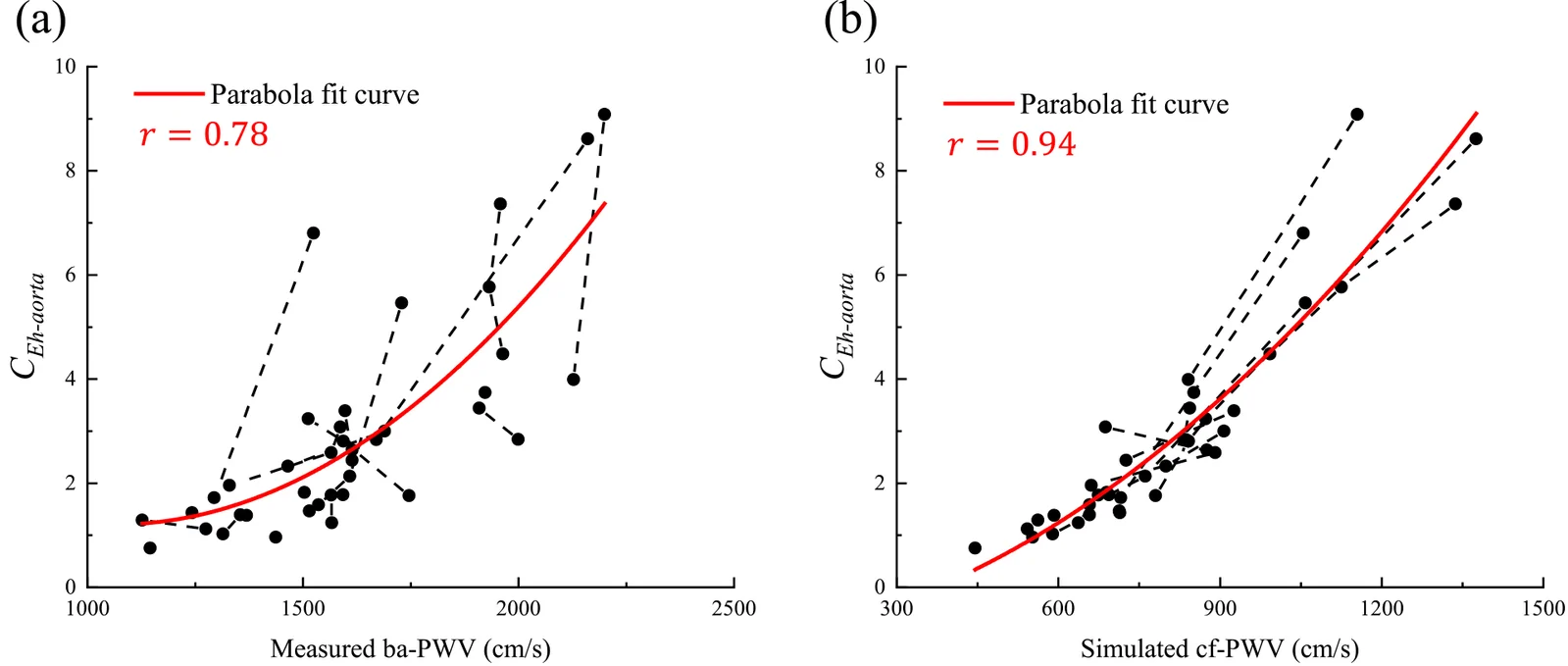
Comparisons of the optimized aortic stiffness coefficient $C_{Eh-\text{aorta}}$ with (a) measured ba-PWV and (b) simulated cf-PWV. Connected points via dashed lines belong to a single patient.

### 3.3. Effects of antihypertensive agents on arterial stiffness

For patients with multiple datasets (k>1), it is possible to track the time-varying arterial stiffness at different arterial locations and investigate the effects of various antihypertensive agents. In figure 7, we plot the variations of arterial stiffness, peripheral resistance and blood pressures for patients with two or three datasets. The patients were classified into three groups according to the agents received: group A received CCBs, group B received ACEIs/ARBs and group C received both CCBs and ACEIs/ARBs. As shown in the figure, CCBs can effectively reduce elevated arterial stiffness in the aorta, carotid and limb clusters. However, when the initial stiffness is low/normal, CCBs have minimal effect. This phenomenon may be owing to the multi-layered structure of the arterial wall. The arterial wall consists of three layers: the tunica intima, tunica media and tunica externa. Smooth muscle cells are distributed in the tunica media, and CCBs may alter their stiffness by blocking calcium ions that flow into the muscle cells. Recent studies [50] have suggested that arterial stiffening is primarily associated with changes in smooth muscle cells. Therefore, we infer that high arterial stiffness is mainly attributable to the elevated stiffness of the smooth muscle layer, while the normal stiffness of healthy arteries is primarily determined by the elastic and collagenous fibres.

**Figure 7.**
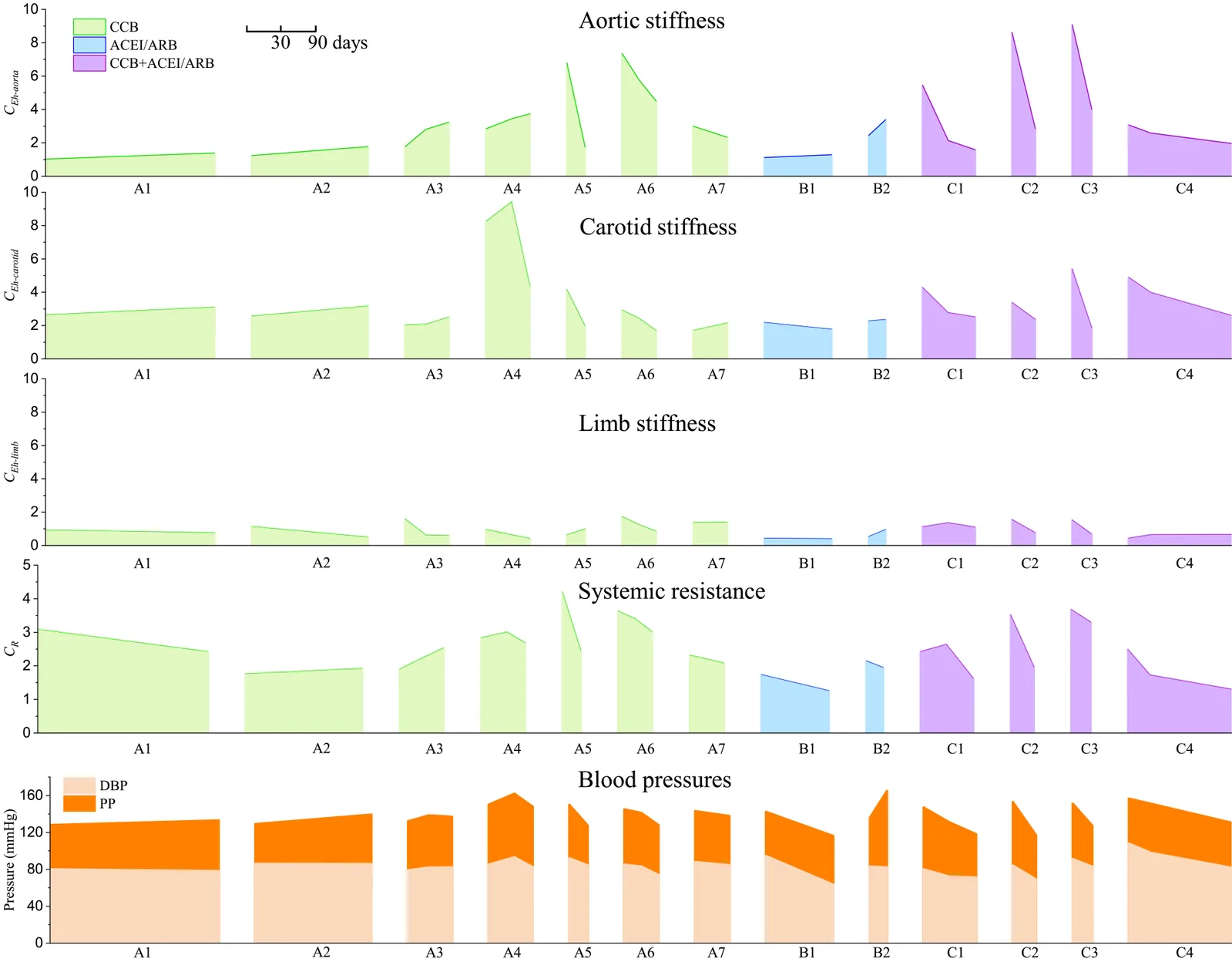
Time-varying key model parameters and blood pressures of patients receiving different antihypertensive agents, with patient ID labelled at the bottom. Group A received CCBs, group B received ACEIs/ARBs and group C received both CCBs and ACEIs/ARBs.

Compared with CCBs, ARB/ACEI agents have a smaller effect on arterial stiffness, likely because these agents primarily reduce peripheral resistance to lower blood pressure. As shown in the figure, ARB/ACEI agents are more effective in reducing $C_{R}$, except for patients B2 and C3, who had been on the medication for a relatively short period. Additionally, CCBs can also reduce elevated $C_{R}$, but their effect is less pronounced for patients with moderate or low $C_{R}$.

The pressure data in figure 7 reveal that significant blood pressure reduction is associated with decreases in key model parameters. In particular, the decrease in diastolic blood pressure is closely associated with the decrease in $C_{R}$. The average change in DBP was –1.5 mm Hg in group A and –14 mm Hg in groups B and C combined, suggesting differential effects between CCBs and ARB/ACEI agents.

## 4. Discussion

This study presents a novel method to infer arterial stiffness based on personalized one-dimensional haemodynamic modelling of the human arterial system, using measured blood flow velocity waveforms and blood pressure values. Unlike widely used PWV-based methods, our approach directly estimates the product of the elastic modulus and thickness of arterial walls. The results suggest that the optimization algorithm converges effectively and demonstrates numerical stability. The simulated ba-PWVs closely align with the measured values, thereby validating the proposed modelling method. By tracking patients using different types of antihypertensive agents, we observed reasonable variations in arterial stiffness over time.

The proposed method offers several advantages compared with existing techniques [12,14,19,25,28] for assessing arterial stiffness. First, it can be implemented at low cost, leveraging widely available ultrasound devices and blood pressure monitors. Another key benefit is that it allows for the assessment of stiffness in deep arteries, whereas PWV measurements typically require superficial arteries and a long distance between measurement points to ensure accuracy.

In clinical practice, selecting appropriate antihypertensive therapy remains challenging, often requiring a trial-and-error approach. The results in §3.3 suggest that our method may assist in guiding the precise use of antihypertensive agents. The major dynamic causes of hypertension include high peripheral resistance and high aortic stiffness. By analysing personalized model parameters, doctors could more accurately select the appropriate type of antihypertensive agents. As shown in figure 8, blood pressures in patients with high aortic stiffness can be effectively controlled by CCBs, whereas CCBs are less effective in patients with normal aortic stiffness. Moreover, the results indicate that the parameter may have diagnostic value. For instance, CCBs may be more beneficial for patients with $C_{Eh-\text{aorta}}>4$ (or $Eh_{\text{aorta}}>1.6\;\text{MPa}\text{mm}$), while ACEIs/ARBs may be preferable in patients without elevated aortic stiffness, as shown in figure 8a. By contrast, the measured ba-PWV does not reliably indicate high aortic stiffness, likely because it is influenced by the stiffness of brachial and femoral arteries. A larger cohort will be required to determine a more accurate and robust critical value. By applying personalized one-dimensional model-guided precision medicine, the control rate of high blood pressure could potentially be further improved.

**Figure 8.**
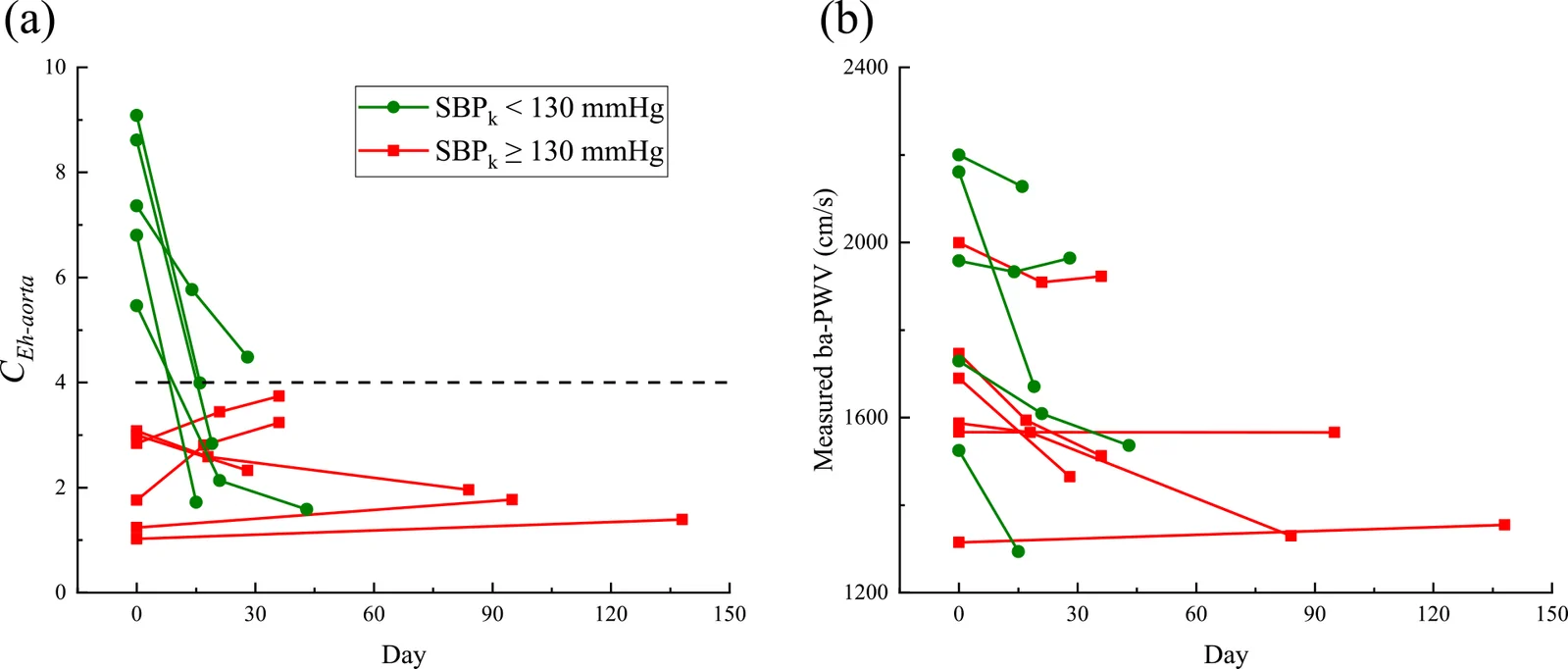
Temporal variations in arterial stiffness indices in patients receiving CCB type antihypertensive agents: (a) the optimized aortic stiffness coefficient $C_{Eh-\text{aorta}}$; (b) measured ba-PWV.

The experiment was limited by the small sample size owing to strict inclusion criteria. To track significant variations in haemodynamic parameters, only hypertension patients who were either starting treatment for the first time or changing their antihypertensive agents were included. Future studies with larger cohorts are needed to further validate the method and its diagnostic potential. Additionally, a key limitation is the absence of direct aortic stiffness measurements for validation. Although less automated than ba-PWV measurements, cf-PWV measurement should be incorporated in future research to enable more direct comparisons between predicted and measured values.

There are also several limitations associated with ultrasound-based measurements: (i) measurement precision depends on the operator’s experience, (ii) measuring the length of an artery and the diameters of all arterial segments is impractical, leading to the use of uniform scaling, which introduces errors, and (iii) acquiring precise Doppler flow waveforms from smaller or deeper arteries is challenging, especially when compared to the common carotid artery. The four-dimensional flow MRI combined with full-body MRI/CT scanning presents a promising alternative for measuring velocity waveforms and geometric data.

The modelling assumptions are also simplified in this study. Equation (2.1*c*) is based on linear elasticity, while nonlinear elasticity exists in real arteries. The blood velocity profile is assumed to be parabolic in each segment, which simplifies real pulsatile flow fields in human arteries. In addition, the inflow boundary conditions are also simplified to piecewise cosine functions owing to the limitations of the Doppler ultrasound images. In future studies, more realistic modelling methods using advanced medical imaging techniques should be investigated.

The personalized one-dimensional modelling method based on inverse analysis of measured flow waveforms can be extended to other applications. For instance, by measuring flow waveforms in each cerebral artery, the stiffness and peripheral resistance of individual cerebral arteries could be identified, enabling more precise evaluation of cerebral arterial function. This method could also be applied to study other arterial regions, such as renal arteries. Furthermore, by tracking time-varying model parameters, the effects of medications targeting the cardiovascular system could be quantitatively evaluated, facilitating precision medicine and drug discovery.

## 5. Conclusion

In this study, we established an inverse modelling framework to quantify arterial stiffness by integrating measured blood flow velocity waveforms and blood pressures with personalized one-dimensional haemodynamic simulations. The one-dimensional haemodynamic model faithfully fitted subject-specific CCA flow waveforms and pressure profiles, and the accompanying optimization procedure demonstrated strong robustness across individuals, with an average m.s.e. of 4.09 ${\text{cm}}^{2}{\text{s}}^{-2}$ for the flow waveforms. Simulated ba-PWV showed good agreement with clinical measurements, indicating that the approach yields reliable estimates of arterial stiffness. We further identified temporal variations in arterial stiffness among patients receiving different antihypertensive agents, with CCB-type therapies effectively attenuating elevated stiffness. Collectively, these findings highlight the potential of this framework as a practical and clinically scalable tool for non-invasive assessment of arterial stiffness.

## Supplementary Material



## Figures and Tables

**Figure 9.**
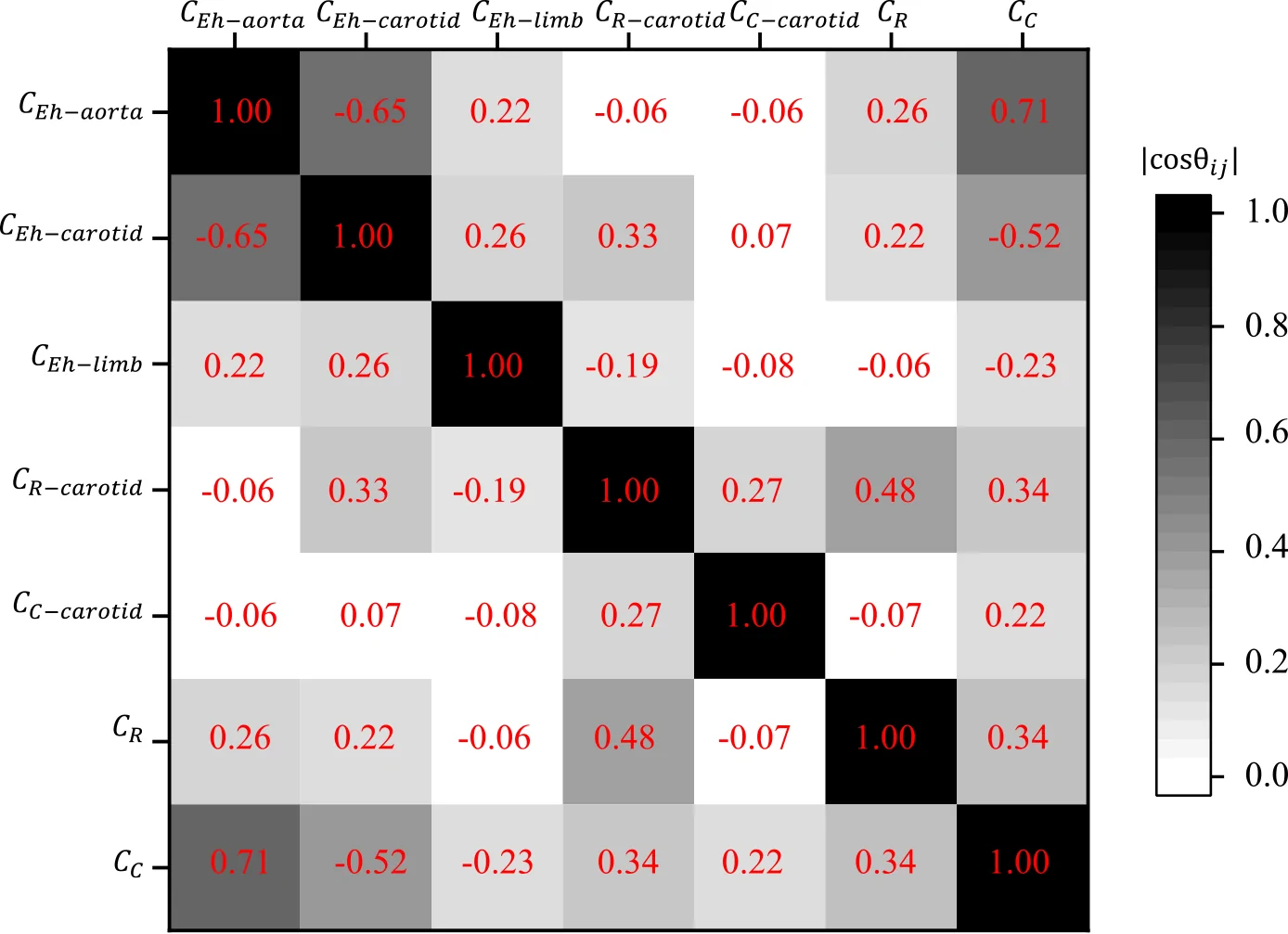
The angles between the perturbed vectors $J_{i}$.

**Figure 10.**
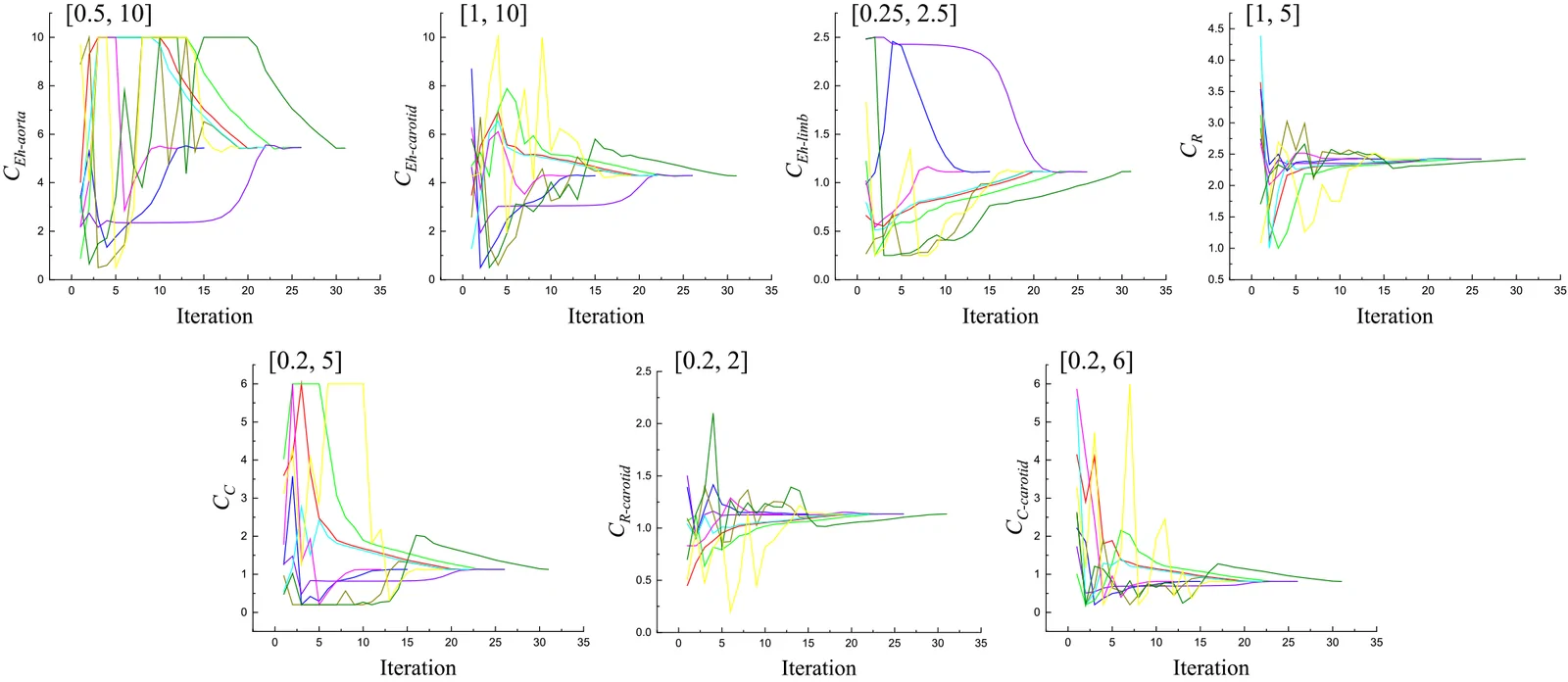
Parameter convergence process of a patient with randomly initialized parameters. $\left[a_{j},b_{j}\right]$ denotes the random sampling interval for the initial parameter values.

## Data Availability

All data can be found in the supplementary material. Supplementary material is available online [51].

## Appendix A. Initial parameters before optimization

The initial parameters for the artery tree are listed in table 3, including the length L, proximal radius $r_{p}$, distal radius $r_{d}$, Young’s modulus E, wall thickness h, connectivity information and the parameters of the coupled outlet zero-dimensional models. The aortic cluster consists of segments 1, 2, 10, 12, 13, 25, 27, 29, 31 and 33. The carotid cluster consists of segments 5, 6, 11, 16, 39, 40, 47 and 48. The limb cluster consists of segments 3, 4, 7, 8, 9, 15, 17, 18, 19, 34–46 and 49–55.

## Appendix B. Uniqueness of the optimized solutions

Although the number of sample points n is significantly larger than the number of parameters, if two parameters change velocity waveforms and blood pressures in a similar or inverse pattern, multiple solutions may exist when solving the inverse problem. To get unique solutions, the columns of the Jacobian matrix should be independent of each other. Here, we use the perturbed vector $J_{i}$ to represent the i-th column of the Jacobian matrix, and we calculate the angle $\theta_{ij}$ between $J_{i}$ and $J_{j}$ to investigate their linear independence:

(B 1) $$\text{cos}\theta_{ij}=\frac{J_{i}\cdot J_{j}}{\left\|J_{i}\|\cdot \|J_{j}\right\|}.$$

If $\text{cos}\theta_{ij}$ approaches 1.0 or –1.0, the two perturbed vectors tend to be linearly dependent, and the two parameters have similar effects on the CCA waveforms and blood pressure. In such situations, multiple solutions may exist. By using the model with default values, we calculated $\text{cos}\theta_{ij}$ between different model parameters. From the matrix in figure 9, we can learn that the maximum absolute value of $\text{cos}\theta_{ij}$ is 0.71 hence there are no significantly correlated parameters. To further verify this, we optimized one case using different initial parameter values, with each parameter randomly sampled from a continuous uniform distribution over $\left[a_{j},b_{j}\right]$. The lower bound $a_{j}$ and the upper bound $b_{j}$ for each parameter $s_{j}$ were chosen to encompass all optimized values obtained across the cohort. The convergence process and the lower and upper bounds for the random sampling are shown in figure 10. As shown in the figure, the randomly initialized parameters eventually converged to a unique solution.
